# What is the role of head computed tomography in post-resuscitation care?

**DOI:** 10.1186/cc13676

**Published:** 2014-03-17

**Authors:** Y Valzani, A Marudi, S Baroni, G De Grandis, R Stacca, E Bertellini

**Affiliations:** 1 University of Modena and Reggio Emilia, Modena, Italy; 2 Nuovo Ospedale Civile Sant’Agostino Estense, Modena, Italy

## Introduction

Guidelines recommend to perform head computed tomography (CT) in nontraumatic cardiac arrest only if the patient remains unconscious after return of spontaneous circulation [1]. Nevertheless, in cardiac arrest due to neurological causes, head CT would dramatically change the therapeutic strategy in these patients [2]. The aim of this study is to present the experience of our ICU in nontraumatic out-of-hospital cardiac arrest (OHCA) patients and to demonstrate that neurological abnormalities can be the primary cause of cardiac arrest.

## Methods

We collected data for all patients with nontraumatic OHCA admitted to the ICU from 1 January 2012 to 31 December 2012. We recorded mean age, male-to-female ratio, cause of cardiac arrest and mortality in the ICU.

## Results

Thirty-four survive cardiac arrest patients were admitted to the ICU. Mean age was 66 years. The male-to-female ratio was 2:1. Sixteen patients died in the ICU. Head CT scans obtained in the immediate postarrest period found significant abnormalities in four patients (three subarachnoid hemorrhage and one ischemic ictus) with immediate modification of treatment.

## Conclusion

Head CT abnormalities can be found in cardiac arrest patients. Further investigations are necessary to evaluate the impact of cardiac arrest from neurological causes and to establish the role of neuroimaging in these patients.
